# An UPLC-ESI-MS/MS Assay Using 6-Aminoquinolyl-*N*-Hydroxysuccinimidyl Carbamate Derivatization for Targeted Amino Acid Analysis: Application to Screening of *Arabidopsis thaliana* Mutants

**DOI:** 10.3390/metabo2030398

**Published:** 2012-07-06

**Authors:** Carolina Salazar, Jenny M. Armenta, Vladimir Shulaev

**Affiliations:** 1 Department of Biological Sciences, College of Arts and Sciences, University of North Texas, 1155 Union Circle No. 305220, Denton, TX 76203, USA; Email: Carolina.Salazar@unt.edu (C.S.); Vladimir.Shulaev@unt.edu (V.S.); 2 Waters Corporation, 100 Cummings Center, Suite 407N, Beverly, MA 01915; Email: jenny_armenta@waters.com

**Keywords:** 6-aminoquinolyl-*N*-hydroxysuccinimidyl carbamate, amino acid analysis, UPLC-ESI-MS/MS, metabolomics, *Arabidopsis thaliana*, mutant screening

## Abstract

In spite of the large arsenal of methodologies developed for amino acid assessment in complex matrices, their implementation in metabolomics studies involving wide-ranging mutant screening is hampered by their lack of high-throughput, sensitivity, reproducibility, and/or wide dynamic range. In response to the challenge of developing amino acid analysis methods that satisfy the criteria required for metabolomic studies, improved reverse-phase high-performance liquid chromatography-mass spectrometry (RPHPLC-MS) methods have been recently reported for large-scale screening of metabolic phenotypes. However, these methods focus on the direct analysis of underivatized amino acids and, therefore, problems associated with insufficient retention and resolution are observed due to the hydrophilic nature of amino acids. It is well known that derivatization methods render amino acids more amenable for reverse phase chromatographic analysis by introducing highly-hydrophobic tags in their carboxylic acid or amino functional group. Therefore, an analytical platform that combines the 6-aminoquinolyl-*N*-hydroxysuccinimidyl carbamate (AQC) pre-column derivatization method with ultra performance liquid chromatography-electrospray ionization-tandem mass spectrometry (UPLC-ESI-MS/MS) is presented in this article. For numerous reasons typical amino acid derivatization methods would be inadequate for large scale metabolic projects. However, AQC derivatization is a simple, rapid and reproducible way of obtaining stable amino acid adducts amenable for UPLC-ESI-MS/MS and the applicability of the method for high-throughput metabolomic analysis in *Arabidopsis thaliana* is demonstrated in this study. Overall, the major advantages offered by this amino acid analysis method include high-throughput, enhanced sensitivity and selectivity; characteristics that showcase its utility for the rapid screening of the preselected plant metabolites without compromising the quality of the metabolic data. The presented method enabled thirty-eight metabolites (proteinogenic amino acids and related compounds) to be analyzed within 10 min with detection limits down to 1.02 × 10^−11^ M (*i.e.*, atomole level on column), which represents an improved sensitivity of 1 to 5 orders of magnitude compared to existing methods. Our UPLC-ESI-MS/MS method is one of the seven analytical platforms used by the Arabidopsis Metabolomics Consortium. The amino acid dataset obtained by analysis of Arabidopsis T-DNA mutant stocks with our platform is captured and open to the public in the web portal PlantMetabolomics.org. The analytical platform herein described could find important applications in other studies where the rapid, high-throughput and sensitive assessment of low abundance amino acids in complex biosamples is necessary.

## 1. Introduction

In the post genomics era where the plant biology community is facing the challenge of identifying the functionalities of genes of unknown function (GUFs), metabolomics offers a link between biochemical phenotype and gene function [1]. However, the use of metabolomics for the prediction of the function of plant genes faces technical challenges due to the large size (between 100,000 to 200,000 biocompounds) [2,3,4], the chemical complexity and the different abundance levels of a plant metabolic pool. These challenges are currently being tackled by combining targeted and non-targeted metabolic analyses to characterize and compare changes in metabolic networks [1,2,5,6,7]. Those combined strategies are a partial solution to the lack of universality of a single analytical technique, as they exploit the power of current separation technologies and the various dynamic ranges and sensitivities offered by the arsenal of commercially available analytical detectors to cover a larger portion of the metabolome than any single platform alone [2,5,6,7,8].

Currently, combined metabolomics technologies are being tested as functional genomic tools for the annotation of *Arabidopsis thaliana* GUFs [1,7,9]. Usually, high throughput biochemical screening methods are employed to first identify previously uncharacterized *Arabidopsis* mutants affecting a variety of metabolic pathways. The screening is carried out by targeted analysis of specific groups of compounds or metabolic subsets (glucosinolates, fatty acids, phytosterols, isoprenoids, amino acids, among others) across a large population of mutagenized *Arabidopsis* lines. Once new loci involved in plant metabolism are identified further work is performed in those particular mutants using non-targeted analysis in order to characterize metabolite changes more broadly. Identification of metabolites that are discriminatory between the knockout plant compared to the wild-type help fill up the gaps in our understanding of plant-specific regulatory and biosynthetic pathways and determine the function of the GUFs [1,7,9].

Because of the central role that amino acids play in plant biochemistry, screening methods that quantify free levels of this class of metabolites in plant tissue are in demand. Despite the numerous methods available for amino acid analysis, many lack the suitability for metabolomic studies. Three aspects are vital in developing an effective targeted metabolite analysis platform for large-scale mutant screening: (i) reduction of sample preparation and analysis time, (ii) collection of high-quality data, and (iii) broad dynamic range [10].

Chromatographic separation methods (gas chromatography, GC, and liquid chromatography, LC) combined with tandem mass spectrometric (MS/MS) detection are dominating the field of metabolomics. Although considerable work has been done in the development of LC-MS methods for analysis of both underivatized and derivatized amino acids in complex matrices, the former are being particularly implemented in metabolomic research and employ the ion-pairing (IP) reversed-phase (RP) LC [10,11] or hydrophilic interaction chromatography (HILIC) alternatives [12,13]. Although these methodologies are very attractive due to the elimination of the sample derivatization step, they suffer of several problems. 

In IPRPLC, for example, the occurrence of system peaks during the gradient elution disturbs the quantitation of amino acids. Systems peaks are caused by low volatility of ion-pairing reagents (example, pentadecafluorooctanoic acid, PDFOA) and their adsorption on the column support surface [14,15]. In addition, long equilibration times (t_e_) between runs and column regeneration after few injections are needed in order to avoid degradation in chromatography and retention time drift for amino acids due to accumulation of the ion-pairing reagent on the column surface. Equilibration times from 9 to 105 min [15,16,17,18] and column flushing from 3 to 30 min are reported in the literature [14,15,19,20]. Another drawback associated with the use of ion-pairing reagents in LC-ESI-MS analysis is the decrease in ionization efficiency of amino acids due to interference by these easy-ionized mobile phase modifiers [21]. The occurrence of undesirable reactions between ion-pairing reagents and salts present in biological samples can also contribute to this problem. Armstrong *et al*. [20] reported the formation of a sodium adduct of tridecafluoroheptanoate (TDFHA) during the analysis of 25 physiological amino acids and one peptide in plasma samples by IPRPLC coupled to time-of-flight (TOF) MS which caused significant signal suppression of alanyl-glutamine dipeptide and valine. A cation-exchange cleanup step had to be added to the sample preparation in order to decrease the abundance of the TDFHA adduct and improve the accuracy and precision of the analysis [20]. Last but not least, surfactant impurities can make the eluent particularly noisy at the m/z range corresponding to underivatized amino acids, affecting the sensitivity of the analysis [15,22].

Alternatively, when HILIC separation mode is used instead of a reversed-phase system underivatized amino acids are retained without any mobile phase modifier and the above mentioned drawbacks associated to the use of iron-pairing reagents can be avoided. Despite of that, column care (*i.e.*, installation of in-line filter and guard column [23]) and long equilibration times (usually about 10 min in order to ensure retention time repeatability [23]) are essential in HILIC analysis. Furthermore, HILIC columns suffer of poor separation efficiency compared to the RPLC technique [24,25].

Due to the above, it is necessary to explore the possibility of implementing LC-MS methods for the analysis of derivatized amino acids to large-scale mutant screening in metabolomic studies. It is undeniable that derivatization brings several advantages to the LC-MS amino acid analysis in complex biological samples. First, derivatization of amino acids improves chromatographic properties (symmetric peak shape, better retention and resolution) in RPLC techniques [22]. In addition, if the amino acid derivatives are amenable for electrospray ionization-tandem mass spectrometry (ESI-MS/MS), better ionization efficiency and increased detection sensitivity can be obtained in their analysis due to the shift of the molecular ion masses and enhanced hydrophobicity caused by derivatization [22]. Despite these advantages it is recognized that not all of the typical amino acid derivatization methods are amenable for “omic”-scale projects.

Typical pre-column derivatization reagents for RPLC amino acid analysis include *o*-phthalaldehyde (OPA), phenylisothiocyanate (PITC), 5-dimethylamino-1-naphthalenesulphonyl-chloride (Dansyl), 9-fluorenylmethyl chloroformate (FMOC), propyl chloroformate (PrCl), butanol, among others. Several disadvantages are associated with these pre-column derivatization methods and the analysis of their derivatives by LC-MS and LC-MS/MS: (i) long derivatization reaction time (Dansyl, 35–50 min [26], PITC, 20 min [27], FMOC, 1 hr [22], Butanol, 1 hr [22]), (ii) complex sample preparation (PITC [27]), (iii) inability to derivatize secondary amino acids (OPA [26]), (iv) derivative instability (OPA [26,28,29]; PITC [30]), (v) photosensitive adducts (Dansyl [28]), (vi) inconsistent production of derivatives (Dansyl [28]), (vii) extraction of excess reagent must be performed to stop derivatization and avoid spontaneous hydrolysis of adducts (FMOC [26,31]), (viii) removal of excess reagent is necessary to avoid rapid RPLC column deterioration (OPA [32], PITC [26,27]) and (ix) long analysis time of amino acid derivatives by LC-MS and LC-MS/MS (20–45 min [22,31,32,33,34]). These disadvantages render these derivatization methods impractical for metabolomics analysis since they introduce errors which can compromise the quality of the data.

The aforementioned shortcomings have urged the development of additional pre-column derivatization reagents. This new generation of reagents has the additional advantage of rendering amino acid adducts with desirable features for LC-MS/MS analysis. These reagents include *N*-hydroxysuccinimide-activated *N*-alkylnicotinic acid esters (Cn-NA-NHS) [35], *p-N,N,N*-trimethyl- -ammonioanilyl *N’*-hydroxysuccinimidyl carbamate iodide (TAHS) [36], 3-aminopyridyl-*N*-hydroxysuccinimidyl carbamate (APDS) [37,38], (5-*N*-succinimidoxy-5-oxopentyl)- triphenylphosphonium bromide (SPTPP) [25], and iTRAQ (isobaric tag for relative and absolute quantitation) [39,40]. Although highly sensitive and selective detection of amino acids is attained by LC-MS/MS when employing these new generation of reagents, unfortunately the reagents are not commercially available (iTRAQ being the exception but it is prohibitively expensive) and some derivatization procedures are still complex and time-consuming. Advantages and shortcomings of these pre-column derivatization methods can be found in the literature [25,35,36,37,38,39,40].

In this study, an analytical platform that combines ultraperformance liquid chromatography with tandem mass spectrometry (UPLC-MS/MS) for targeted amino acid analysis in *Arabidopsis thaliana* leaf extracts is presented. Our method uses the commercially available amino acid derivatization reagent 6-aminoquinolyl-*N*-hydroxysuccinimidyl carbamate (AQC). Since its introduction as derivatization reagent, AQC has shown interesting features. Reaction of AQC with primary and secondary amino acids is a simple, straightforward process that occurs within seconds and produces stable derivatives; in contrast the hydrolysis of the excess reagent is a much slower reaction [30,41]. The only disadvantages reported in the literature are related to the use of HPLC separation with fluorescence or UV detection: long analysis time (25–65 min), low sensitivity (UV only), peak interference by AQC hydrolysis product and intramolecular quenching [41,42,43,44,45]. An analytical platform that exploits the greater chromatographic capacity and throughput of UPLC and the sensitivity and selectivity of MS/MS would overcome those drawbacks. The applicability of a UPLC-MS/MS method coupled with AQC precolumn derivatization for targeted amino acid analysis in large-scale metabolomics studies is demonstrated.

## 2. Results and Discussion

### 2.1. Development of an Infusion Protocol for ESI-MS/MS Parameter Optimization of AQC Amino Acid Derivatives

Derivatization with AQC offers a simple and reproducible conversion of amino acids into their stable adducts amenable for RPLC [41]. Although the superior throughput and resolution of the UPLC technology can now be combined with UV, fluorescence (FL), or photodiode array (PDA) detection of AQC amino acid derivatives thanks to the commercial availability of the AccQ•Tag Ultra Chemistry package (Waters Corp.) [46,47], the possibility of using UPLC-MS technology has not received enough attention even though the eluents used for AccQ•Tag UPLC amino acid analysis and the AQC adducts are amenable for MS. Armstrong *et al*. [20] pointed out that although the preparation of samples for LC-MS analysis using amino acid kits simplifies the derivatization step, the non-volatile buffers included in those kits (such as the Waters AccQ•Tag) are not readily compatible with ESI-MS, bringing disadvantages to the LC-MS approach.

Our preliminary studies in the optimization of MS parameters for the analysis of amino acids derivatized with the AccQ•Tag kit proved that signal suppression was particularly problematic during direct infusion of the adducts into the mass spectrometer. As indicated by Armstrong *et al*. [20], this problem is attributed to the non-volatile borate buffer provided with the AccQ•Tag derivatization kit, which is used for optimum pH adjustment of the reaction solution in order to obtain maximum product yields [41].

To overcome the drawback presented by the borate buffer in direct infusion experiments, an alternative buffer for the AQC derivatization of amino acids is needed in order to facilitate the optimization of critical MS parameters (cone voltage and collision energy) that affect the selectivity and sensitivity of LC-MS/MS amino acid analysis.

#### 2.1.1. Evaluation of the Effect of Buffer System Type, pH and Concentration on the AQC-Amino Acid Derivatization

In order to find a suitable alternative buffer for AQC amino acid derivatization, several factors affecting the outcome of the reaction, such as the effect of the chemical nature, concentration, and pH of the reaction medium were investigated. Two MS friendly volatile buffers, namely, ammonium formate, and ammonium acetate were studied. For comparison purposes, control experiments using the well established borate buffer system (pH 8.8) as the reaction medium were carried out in parallel. An initial judgment on the suitability of the media under evaluation was made based on the physical appearance of their respective amino acid standard solutions following derivatization with AQC. The use of ammonium formate buffer (pH 7.6) produced dark-yellowish solutions upon AQC amino acid derivatization, possibly indicating the formation of unwanted byproducts. The ammonium acetate buffer (pH 9.3), on the other hand, yielded clear colorless solutions similarly to the borate buffer system and was selected for further experiments. 

The effect of the buffer concentration on the derivatization reaction was investigated next, while keeping the pH constant at 9.3. Six concentrations of ammonium acetate buffer (10, 20, 50, 100, 200 and 500 mM) were tested. All six concentrations yielded clear colorless solutions upon AQC amino acid derivatization. Nevertheless, subsequent UPLC-ESI-MS/MS analysis revealed a decrease in ion intensity with the increase in buffer concentration. Evidently, high buffer concentrations led to an increase in salt deposits in the sample cone surface, decreasing the signal intensity. Signal intensity was particularly affected at buffer concentrations 100 mM and higher. The increased LC-MS/MS signal suppression with increasing buffer concentration has been reported by other authors [48]. Ammonium acetate buffer concentrations equal or less than 50 mM did not show significant signal suppression and were found appropriate for AQC amino acid derivatization.

Using a constant ammonium acetate buffer concentration of 50 mM, the pH was then adjusted to 9.0, 9.3 and 10.3. Buffered amino acid solutions at pH 9.0 turned slightly yellowish upon AQC derivatization. At pH 9.0, lowering the buffer concentration from 50 mM to 20 mM produced even darker yellowish solutions, further indicating that both the pH and the buffer concentration affect AQC amino acid derivatization. Ammonium acetate buffer concentrations greater than 50 mM at the pH of 9.0 were not tested based on our previously results, showing a decrease in ion intensity with an increase in buffer concentration. Keeping derivatization conditions at pH = 10.3 also proved suitable for AQC adduct formation, and no differences were observed compared to the results obtained at pH 9.3 (data not shown). All further infusion experiments were performed using the 50 mM ammonium acetate buffer system at pH 9.3.

#### 2.1.2. Evaluation of Derivative Stability and Reproducibility of the AQC Derivatization Reaction Using the Alternative 50 Mm Ammonium Acetate Buffer (pH 9.3)

The applicability of the 50 mM ammonium acetate buffer (pH 9.3) in the preparation of AQC amino acid derivates for direct infusion experiments was evaluated. Derivatized amino acid standard solutions (1 × 10^−2 ^g/L) were infused into the Xevo TQ mass spectrometer. Multiple reaction monitoring (MRM) transitions were determined for 26 amino acids, and the optimal cone voltage and collision energy associated with each transition were established (Table 1). Unlike previous direct infusion experiments performed with the borate buffer, signal suppression and source contamination were not observed with this alternative buffer system, after 78 consecutive infusions. AQC amino acid derivatives were stable for more than three weeks when stored at room temperature in the dark, further advocating the effectiveness of this buffer for the derivatization reaction (data not shown).

**Table 1:** MRM transitions, cone voltage (CV) and collision energy (CE) determined for AQC-derivatized standard amino acids buffered with ammonium acetate (50 mM, pH 9.3). Experimental conditions: Waters XEVO TQ mass spectrometer; direct infusion at 20 µL/min; final amino acid concentration after derivatization was 1 × 10^−2^ g/L.

Compound	Parent ion	Daughter ion	CV (V)	CE (eV)
**β-Alanine**	260.1	171.0	27	21
**L-Alanine**	260.2	171.0	25	21
**L-Asparagine**	303.1	171.0	24	21
**L-Aspartic acid**	304.1	171.0	27	23
**L-Arginine**	345.2	171.0	27	17
**L-Citrulline**	346.2	171.0	22	24
**L-Cysteine**	292.1	171.0	27	21
**L-Cystine**	411.4	171.0	20	18
**L-Glutamic acid**	318.1	171.0	27	21
**L-Glutamine**	317.1	171.0	22	24
**L-Glycine**	246.1	171.0	27	21
**L-Histidine**	326.1	171.0	18	12
**L-Hydroxy-L-Proline**	302.1	171.0	24	21
**L-Isoleucine**	302.2	171.0	28	21
**L-Leucine**	302.2	171.0	27	20
**L-Lysine**	317.2	171.0	18	18
**L-Methionine**	320.1	171.0	27	21
**L-Ornithine**	303.2	171.0	16	18
**L-Phenylalanine**	336.1	171.0	29	21
**L-Proline**	286.1	171.0	23	21
**L-Serine**	276.1	171.0	25	19
**L-Taurine**	296.1	171.0	18	15
**L-Threonine**	290.1	171.0	25	20
**L-Tryptophan**	375.2	171.0	30	25
**L-Valine**	288.2	171.0	28	21

**Figure S1:**
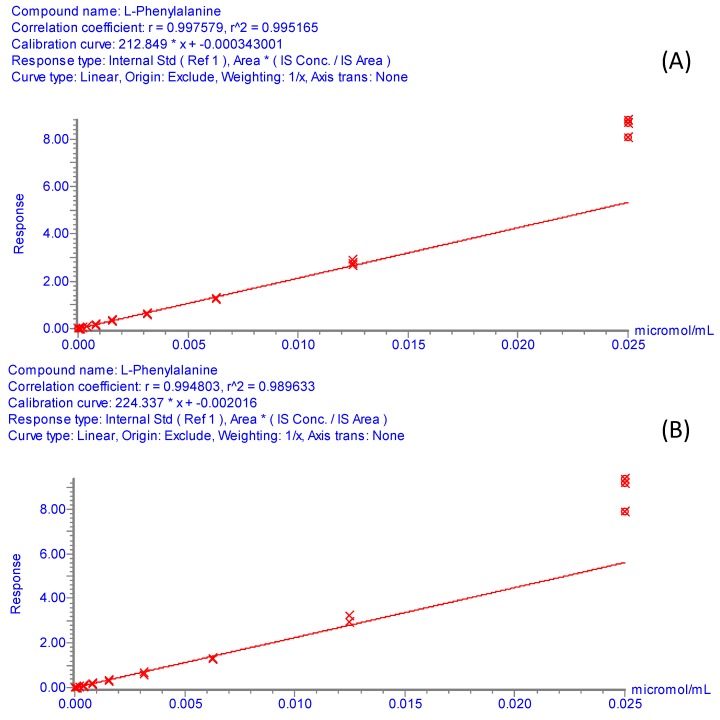
Internal calibration curves for phenylalanine. Experimental conditions: Standard solutions of phenylalanine covered the concentration range from 2.5 × 10^−5^ M to 4.77 × 10^−11 ^M, linear dynamic range observed from 1.25 × 10^−5^ M to 1.22 × 10^−8 ^M. Internal standard (4-Hydroxyphenyl-2,6-d_2_-alanine-2-d_1_-01) at 4 × 10^−4 ^g/L. 1 μL of sample was injected. UPLC-ESI-MS/MS analyses were performed as described in Section 3.5. (A) Phenyl alanine and its internal standard were derivatized with AQC using the borate buffer system. (B) Derivatization with AQC in 50mM ammonium acetate (pH 9.3) as the buffering media.

The reproducibility of the derivatization method with the 50 mM ammonium acetate buffer (pH 9.3) was confirmed by the UPLC-ESI-MS/MS analysis. The peak area of the isotopically labeled amino acids derivatized with AQC in ammonium acetate medium was measured in nine replicates (final concentration of adducts = 4 × 10^−4 ^g/L) (Table S1). As shown in Table S1, the relative standard deviation (RSD) of the peak area for all isotopically labeled amino acids was below 9%, indicating high reproducibility of the derivatization reaction. The efficiency of the reaction in the alternative buffer was further studied by evaluating the linearity of the detector response for standard amino acid solutions over the concentration range from 250 μM to 3.05 pM. Figure S1A and Figure S1B (supplementary information) show typical internal calibration curves of phenylalanine obtained by UPLC-ESI-MS/MS analysis under the conditions described in section 3.5. The response factors for these calibration curves were calculated using relative peak areas, in which the area of phenylalanine was divided by the area of the internal standard, 4-hydroxyphenyl-2,6-d_2_-alanine-2-d_1_ (present at a constant concentration of 4 × 10^−4^ g/L after derivatization). Figure S1A displays the internal calibration curve for phenylalanine obtained with the conventional borate buffer, whereas Figure S1B shows the internal calibration curve obtained with the alternative 50 mM ammonium acetate buffer (pH 9.3). Clearly, both internal calibration curves exhibit similar response factors, correlation coefficients and slopes, providing additional evidence for the suitability of the ammonium acetate buffer for AQC derivatization of amino acids. It should be mentioned, however, that when the calibration curves were built using absolute peak areas, rather than relative peak areas, the overall peak areas measured with the ammonium acetate buffer were lower than those measured with the borate buffer. One plausible explanation for this observation is that although both borate and ammonium acetate buffers are suitable for the reproducible formation of stable AQC amino acid adducts, lower yields of these derivatives are attained with the ammonium acetate buffer system.

In summary, our results show that 50 mM ammonium acetate buffer (pH 9.3) can be effectively used for AQC amino acid derivatization in direct infusion experiments. The use of this alternative buffer allowed the optimization of mass spectrometric parameters specific for AQC derivatized amino acids (such as cone voltage and collision energy) necessary for LC-MS/MS method development, which could not be otherwise obtained with the borate buffer system (Table 1).

At this point, it is worth mentioning, that signal suppression phenomenon observed with borate buffered amino acid derivatives during direct MS infusion experiments was not manifested during their UPLC-ESI-MS/MS analysis. This is mainly because during UPLC analysis the sample itself undergoes dilution with the mobile phase. Therefore, the ammonium acetate buffer simply offers an MS friendly alternative medium for direct MS infusion experiments in order to optimize MS parameters necessary for AQC amino acid derivative analysis (a one-time process necessary for method development). The ion suppression observed with the borate buffer during direct infusion of AQC amino acid adducts should not prevent us for combining a rugged derivatization chemistry such as AccQ•Tag Ultra method and the LC-ESI-MS/MS analytical approach, especially in metabolomics applications where the gain in sensitivity and specificity offered by the MS analysis (in the MRM mode) of derivatized amino acids is highly desirable. Therefore, once these MS parameters are optimized, the specific derivative chemistry of the AccQ•Tag kit is used (*i.e.*, using the borate buffer) for the derivatization step previous to the UPLC-ESI-MS/MS analysis of amino acids in the Arabidopsis mutants. As it was mentioned before, using a derivatization kit that is commercially available is preferred because it simplifies the derivatization step, but most importantly, the specific chemistry of the AccQ•Tag kit offers higher yields of amino acid adducts; both necessary factors for the aim of large scale metabolomics projects.

### 2.2. AccQ•Tag UPLC-ESI-MS/MS Method Development and Evaluation

In our experiments, the UPLC-ESI-MS/MS determination of AQC derivatives of 38 amino acids and 15 labeled internal standards was achieved by operating the mass spectrometer in the MRM mode. The main product from the collision-induced dissociation of all AQC adducts was the ion m/z 171, derived from the cleavage at the ureide bond formed upon derivatization. Therefore, the MRM-MS method was developed to include the transition m/z [M + H]^+^ > 171 for each derivatized amino acid at the corresponding optimized collision energy and cone voltage (Table 1). To increase the overall performance, the MRM-MS method was built to monitor only one amino acid transition per timed function (time windows ranging from 0.42 to 1.03 min).

Although the tandem mass spectrometer provides excellent specificity when operated in the MRM mode, complete resolution of chromatographic peaks corresponding to isomers, isobars and/or isotopomers is desirable for satisfactory quantitation of amino acids in their native or derivatized form [14,19,22,49]. In our study the AccQ•Tag Ultra column, under the gradient conditions described in section 3.5, performed very well and provided good chromatographic resolution for unequivocal peak identification by MS/MS analysis of AQC amino acid derivatives. All the targeted compounds (38 amino acids) and their respective internal standards (15 labeled amino acids) were resolved within 10 min.

The improvement in sample throughput and chromatographic separation brought by UPLC to the analysis of AQC derivatized amino acids was also previously demonstrated by Boogers *et al*. [46] in their UPLC-PDA method. In their comparative study, 16 amino acids were separated within 8 min (total cycle time = 10 min), which resulted in a reduction in time analysis by a factor of 2.5 compared to the Pico•Tag method (a kit from Waters Corporation which uses the PITC as derivatization reagent). In our study a larger number of amino acids were analyzed without compromise in the separation. 

**Figure 1:**
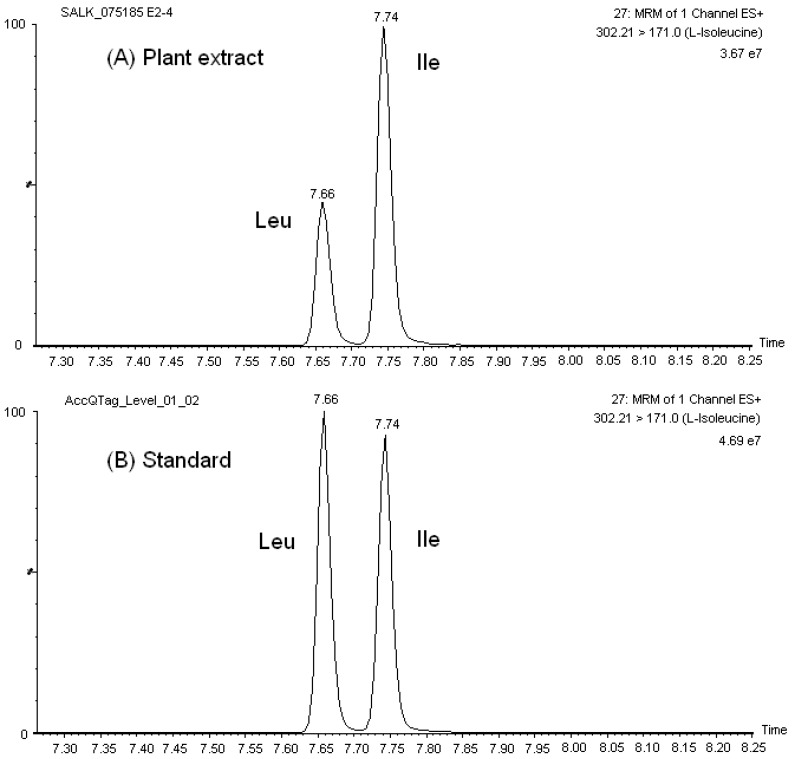
Mass chromatographs of the isobaric set Leu/Ile in (**A**) *A. thaliana* extract, and (**B**) calibration solution (25 μM).

**Figure S2:**
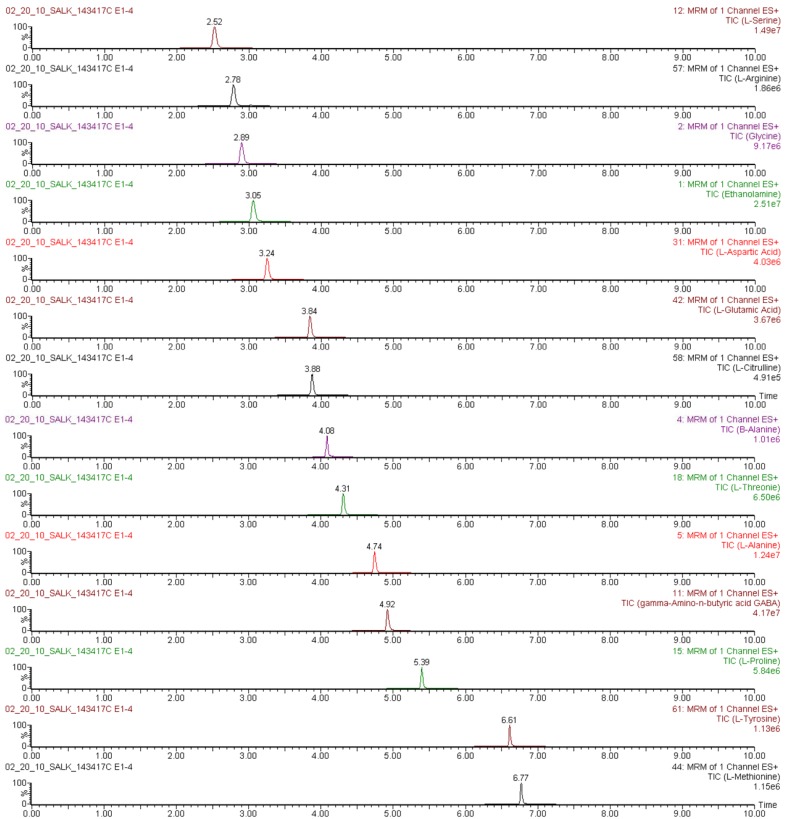
Mass chromatograms of AQC-derivatized amino acids quantified in an Arabidopsis thaliana leaf extract, obtained by UPLC-ESI-MS/MS in multiple reaction monitoring mode.

Our chromatographic method discriminated among the isobaric and/or isomeric sets, namely, leucine (Leu)/isoleucine (Ile)/hydroxyproline (HPro), glutamine (Gln)/lysine (Lys), 1-methylhistidine (1-Mehis)/3-methylhistidine (3-Mehis), threonine (Thr)/homoserine (Hser), sarcosine (Sar)/L-alanine (L-Ala)/β-Alanine (β-Ala), and β-aminoisobutyric acid (Baiba)/α-amino-n-butyric acid (Abu)/γ-amino-n-butyric acid (Gaba). Similarly, the sets glutamine (Gln)/glutamic acid (Glu) and asparagine (Asn)/aspartic acid (Asp) had a very distinguished chromatographic retention. Figure 1 shows the mass chromatograms of the amino acid set Leu/Ile in both standard solutions and Arabidopsis leave extracts. Typical UPLC-ESI-MS/MS mass chromatograms of other amino acids in *A. thaliana* extracts are presented in Figure S2.

Others authors [10,11,49] have reported problems separating and quantifying some of these problematic amino acid sets in their underivatized form using HPLC-MS/MS. Jander *et al*. [11], for example, could not differentiate between Ile/Leu, and unsatisfactory resolution between Lys/Gln adversely affected quantitation in Arabidopsis seed extracts since the tail of the considerably more abundant Gln peak masked the signal from Lys. Using the ion pairing approach, Gu *et al*. [10] reported irreproducible chromatographic resolution of Glu/Gln and Asp/Asn, and, therefore, the contributions of the naturally occurring heavy isotopomer of Asn to the Asp channel and Gln to the Glu channel had to be evaluated. Additionally, although the authors could separate Ile and Leu when standard solutions were analyzed, the amino acid pair was only partially resolved in Arabidopsis seeds extracts. In their same set of experiments, the pair Thr and HSer always coeluted during chromatography regardless of the type of solution analyzed. To solve these problems, Gu *et al*. [10] used alternative MRM transitions for these pairs, but they were not as specific and sensitive for the respective amino acids as the transitions involving the most abundant fragment ions (for example refer to Figure 3 in ref [10]). It was demonstrated before by Petritis *et al*. [49] that selection of less abundant fragment ions caused a four- to six-fold loss of sensitivity for the LC-MS/MS analysis of native amino acids. It is worth noting that the lack of baseline separation [22] and irreproducible amino acid separation between standards and biological samples [36] have also been observed in the HPLC-ESI-MS/MS analysis of amino acids derivatized with FMOC, butanol, PrCl [22] and TAHS [36].

As a result of the reproducible and satisfactory chromatographic separation of AQC amino acid derivatives obtained with the AccQ•Tag Ultra column in our studies, the development of our MRM-MS method was not complicated by overlapping elution of critical sets of amino acids, in contrast to previous observations with HPLC separation of native and derivatized amino acids. For example, it was not necessary to account for the crosstalking of ^13^C isotopes of Asn and Gln to the MRM channels of Asp and Glu, respectively, in order to accurately quantify these amino acids. Furthermore, there was no need to select additional fragment ions for the isomers/isobars, which may be less predominant and decrease the sensitivity of the transition channel used for MS detection of the corresponding amino acid. Additionally, reproducible chromatographic separation was obtained in both amino acid standard solutions and sample extracts (see Figure 1 for example) and, therefore, quantitation of amino acids was straightforward. 

According to these results, the combination of AQC pre-column derivatization with the superior performance of UPLC technologies allows reproducible separation of several critical amino acid pairs before MS/MS analysis, which is a necessity because of their similar nominal masses or identical fragmentation. This adds maximum selectivity and sensitivity to the amino acid analysis.

#### 2.2.1. Method Evaluation

The performance of the UPLC-ESI-MS/MS method for the analysis of AQC-derivatized amino acids was evaluated by measuring the repeatability, linearity, and sensitivity of the analysis. The repeatability of the method was determined by examination of the retention time and peak area ratios (*i.e.*, Area _amino acid_ /Area _internal standard_) after intraday injections of standard solutions of derivatized amino acids. The relative standard deviations (RSDs, in %) of the retention times were always less than 2% (n = 30) for the AQC-amino acids (Table 2 and Table S2). RSD values for peak areas ranged from 0.19 to 7.47% (Table 2). These results compare well with the precision studies obtained for the HPLC-ESI-MS analysis of AQC derivatized amino acids performed by Hou *et al*. [50]. With their method, the RSD% of the peak area ratios was in the range of 1.1 to 4.0% using a mixed standard of 17 AQC-amino acids at the concentration of 100 μM (n = 6). Repeatability of retention time was not given in their study.

**Table 2:** Representative retention time (R_t_) and peak area relative standard deviation (RSD) values obtained from the UPLC-ESI-MS/MS analysis of AQC-derivatized amino acids. Average R_t_ and respective RSD values calculated in standard solutions (n = 30). Average peak area and respective RSD values calculated in the standard solution 2.5 × 10^−5^ M (n = 3).

Amino acid	Retention Time		Peak area	
	Average (min)	RSD (%)	Average	RSD (%)
Hydroxyproline	1.49	1.04	512021.09	2.37
Histidine	1.60	1.22	105030.26	2.32
Asparagine	1.84	0.71	709289.60	0.57
3-Methyl-histidine	1.97	0.83	97257.23	2.03
Taurine	2.13	0.57	446356.38	1.49
1-Methyl-histidine	2.20	0.71	139048.91	0.70
Serine	2.51	0.46	779643.10	0.71
Glutamine	2.67	0.46	691927.17	0.88
Carnosine	2.74	0.45	147176.08	7.47
Arginine	2.77	0.58	220889.30	6.14
Glycine	2.88	0.35	1000873.21	0.40
Homoserine	3.02	0.34	1916733.96	0.24
Ethanolamine	3.04	0.31	2086602.79	0.46
Aspartic acid	3.24	0.32	385270.07	0.58
Sarcosine	3.68	0.19	998421.40	1.27
Glutamic Acid	3.84	0.25	487526.98	0.90
Citrulline	3.87	0.22	362040.92	0.77
β-Alanine	4.08	0.19	1574830.71	1.26
Threonine	4.30	0.15	959005.21	1.40
L-Alanine	4.74	0.15	1130325.08	2.07
γ-Amino-n-butyric acid	4.92	0.10	1973450.25	2.26
α-Amino adipic acid	5.13	0.13	265662.16	2.29
β-Aminoisobutyric acid	5.38	0.16	1618634.88	2.53
Proline	5.39	0.10	1125903.54	2.03
α-Amino-n-butyric acid	5.99	0.10	1274348.50	0.80
Tyrosine	6.61	0.10	876164.79	0.24
Methionine	6.77	0.09	1071936.96	1.52
Valine	6.91	0.04	1252590.04	0.45
Leucine	7.67	0.04	1333786.63	0.19
Isoleucine	7.75	0.02	1345367.13	0.57
Phenylalanine	7.86	0.03	1191579.54	1.16
Tryptophan	7.96	0.05	517505.16	1.07

It is important to point out that the excellent stability of the retention time was observed in our study with injection of calibration standards and *Arabidopsis* extracts without any particular column care, indicating the advantage of our technique over the ion pairing approach in terms of repeatability of the method. Table S2 shows the repeatability of the retention time at two different time points within the chromatographic column lifetime. Retention time shifts were lower than 0.06 min. In the iron pairing approach, retention time migration of underivatized amino acids after a few consecutive assays is especially problematic due to accumulation of the ion-pairing reagent on the surface of the column material [19,20]. Retention time shift for native amino acids of as much as 1 or 1.5 min has been reported in the literature for IPRPLC-MS based studies [19,20]. Therefore, although intra-day RSD values for HPLC retention times found by IPRPLC-MS/MS methods could prove comparable to the values reported in this study (for example, > 3.8% [17], > 1.3% [10]), caution must be exercised when doing a direct comparison since, in some cases, retention time stability, and therefore, reproducible amino acid separation in IPRPLC-MS/MS approaches is contingent to frequent column flush with pure organic solvent after few assays.

The evaluation of the method was continued with data collection from the analysis of twenty solutions containing 38 derivatized physiological amino acids with a concentration ranging from 25 μM to 48 fM and 15 stable-isotope-labeled amino acids at a fixed concentration of 4 × 10^−4^ g/L. The data was used to create an internal calibration curve for each amino acid using the respective internal standard as given in Table S3. Using the internal standardization method, plots of relative peak area *versus* amino acid concentration were generated using the TargetLynx software and were used to calculate the linearity (correlation coefficient and dynamic range) and detection limits shown in Table 3. Linear regression analysis of the calibration curves showed correlation coefficients (R^2^) between 0.9810 and 1.000, within the low and high limits of linearity specific for each amino acid, as presented in Table 3.

**Table 3:** Evaluation results for linearity and sensitivity of UPLC-ESI-MS/MS method for the analysis of AQC-derivatized amino acids.

Amino acid	High limits of linearity (M)	Low limits of linearity (M)	Dynamic range	Correlation coefficient (R^2^)	Detection limit (M)
Hydroxy-L-Proline	2.50 × 10^−5^	1.53 × 10^−9^	10000	0.9970	4.86 × 10^−11^
Histidine	6.25 × 10^−6^	1.22 × 10^−8^	100	0.9972	9.92 × 10^−9^
Asparagine	2.50 × 10^−5^	1.22 × 10^−8^	1000	0.9994	3.73 × 10^−9^
3-Methyl-histidine	1.25 × 10^−5^	2.44 × 10^−8^	1000	0.9950	2.16 × 10^−11^
Taurine	2.50 × 10^−5^	1.53 × 10^−9^	10000	0.9961	1.09 × 10^−11^
1-Methyl-histidine	2.50 × 10^−5^	6.10 × 10^−9^	10000	0.9934	1.02 × 10^−11^
Serine	2.50 × 10^−5^	1.22 × 10^−8^	1000	0.9975	7.42 × 10^−9^
Glutamine	2.50 × 10^−5^	1.95 × 10^−7^	100	0.9983	1.06 × 10^−8^
Carnosine	6.25 × 10^−6^	6.10 × 10^−9^	1000	0.9902	4.13 × 10^−11^
Arginine	2.50 × 10^−5^	1.22 × 10^−8^	1000	0.9997	2.41 × 10^−10^
Glycine	2.50 × 10^−5^	4.88 × 10^−8^	1000	0.9991	3.21 × 10^−9^
Homoserine	3.13 × 10^−6^	1.53 × 10^−9^	1000	0.9810	1.60 × 10^−10^
Ethanolamine	1.25 × 10^−5^	2.44 × 10^−8^	1000	0.9976	2.66 × 10^−9^
Aspartic acid	2.50 × 10^−5^	2.44 × 10^−8^	1000	0.9999	3.17 × 10^−9^
Sarcosine	2.50 × 10^−5^	1.53 × 10^−9^	10000	0.9983	4.83 × 10^−10^
Glutamic Acid	2.50 × 10^−5^	2.44 × 10^−8^	1000	0.9970	4.66 × 10^−9^
Citrulline	2.50 × 10^−5^	6.10 × 10^−9^	10000	0.9956	2.87 × 10^−10^
β-Alanine	1.25 × 10^−5^	1.22 × 10^−8^	1000	0.9999	1.43 × 10^−9^
Threonine	2.50 × 10^−5^	1.22 × 10^−8^	1000	0.9957	1.30 × 10^−9^
L-Alanine	2.50 × 10^−5^	4.88 × 10^−8^	1000	0.9983	1.06 × 10^−9^
γ-Amino-n-butyric acid	1.25 × 10^−5^	2.44 × 10^−8^	1000	0.9981	2.69 × 10^−9^
α-Amino adipic acid	2.50 × 10^−5^	1.22 × 10^−8^	1000	0.9992	9.28 × 10^−11^
β-Aminoisobutyric acid	6.25 × 10^−6^	6.10 × 10^−9^	1000	0.9976	7.18 × 10^−11^
Proline	2.50 × 10^−5^	2.44 × 10^−8^	1000	0.9997	1.55 × 10^−9^
α-Amino-n-butyric acid	2.50 × 10^−5^	1.22 × 10^−8^	1000	0.9991	1.44 × 10^−9^
Tyrosine	2.50 × 10^−5^	1.53 × 10^−9^	10000	0.9925	8.05 × 10^−11^
Methionine	2.50 × 10^−5^	2.44 × 10^−8^	1000	0.9997	1.60 × 10^−9^
Valine	2.50 × 10^−5^	1.22 × 10^−8^	1000	1.000	8.25 × 10^−10^
Leucine	2.50 × 10^−5^	1.53 × 10^−9^	10000	0.9997	2.74 × 10^−10^
Isoleucine	2.50 × 10^−5^	1.53 × 10^−9^	10000	0.9998	1.20 × 10^−10^
Phenylalanine	2.50 × 10^−5^	1.22 × 10^−8^	1000	1.000	6.28 × 10^−10^
Tryptophan	2.50 × 10^−5^	1.53 × 10^−9^	10000	0.9986	6.18 × 10^−10^
δ-Hydroxylysine	1.56 × 10^−6^	4.88 × 10^−8^	100	0.9987	3.57 × 10^−11^
Cystathionine	2.50 × 10^−5^	3.91 × 10^−7^	100	1.000	1.35 × 10^−9^
Ornithine	2.50 × 10^−5^	2.44 × 10^−8^	1000	0.9993	6.77 × 10^−9^
Cystine	6.25 × 10^−6^	3.91 × 10^−7^	10	0.9962	5.93 × 10^−9^
Lysine	1.56 × 10^−6^	1.22 × 10^−8^	100	0.9974	1.50 × 10^−9^
Homocystine	6.25 × 10^−6^	2.44 × 10^−8^	100	0.9979	6.43 × 10^−11^

In addition, the overall process efficiency was calculated as PE (%) = 100 (area _spiked before extraction_/area _standard solution_) as described in Gu *et al*. [10]. The area _standard solution_ corresponds to the area of the internal standard in the neat standard solutions used to prepare the calibration curves, and area _spiked before extraction_ corresponds to the peak area in the sample extract. The overall process efficiencies ranged from 65.0 to 99.4%. As stated by Gu *et al*. [10], process efficiencies greater than 100% occurs when coeluting species present in the sample matrix contribute to the detected signal of the amino acid. There was no evidence of such contribution according to the results presented in Table S4.

Subsequently, the limits of detection (LOD) were established by using the method of the blank and were calculated as three times the standard deviation of the peak areas observed from the blank signals, divided by the slope of the calibration curve obtained for the given amino acid. The LOD values obtained (Table 3) ranged from 1.02 × 10^−11^ to 1.06 × 10^−8^ M, suggesting that the analytical method presented in this study is 1 to 5 orders of magnitude more sensitive than other existing LC-MS and LC-MS/MS approaches [14,15,17,18,21,22,36,37,38,39,49,50,51,52,53] for the analysis of native or derivatized amino acids, as showed in Table 4. The LOD values reported by Shimbo *et al*. [37] for 20 amino acids derivatized with TAHS are comparable to those obtained in our study, however, our UPLC analysis for AQC-amino acids has higher throughput (38 amino acids and 15 internal standards separated three times faster). Table 4 also shows the faster separation time (5 times shorter chromatographic run) and better sensitivity (3 orders of magnitude lower LOD) added to the analysis of AQC-amino acids by the combination of UPLC with tandem mass spectrometry operated in the MRM mode.

**Table 4:** Comparison on the sensitivity of selected LC/MS-based approaches for amino acid analysis.

Method	Derivatization Reagent	AT, min	TCT, min	LOD_conc. based_, M	LOQ_conc. based_, M	Ref.
				(LOD_on column_)	(LOD_on column_)	
IPRPLC-MS	None	27	Unavailable*	3.0 × 10^−6^–1.3 × 10^−5^	--	[ 14]
				(3.5–7.0 pmol)		
IPRPLC-MS	None	40	60	1.2 × 10^−6^–3.4 × 10^−5^	--	[ 15]
IPRPLC-MS/MS	None	20	Unavailable*	3.0 × 10^−8^–4.0 × 10^−6^	--	[ 49]
				(0.3 to 40 pmol)		
IPRPLC-MS/MS	None	20	Unavailable*	6.1 × 10^−7^–6.6 × 10^−6^	--	[ 51]
IPRPLC-MS/MS	None	16	31	--	(0.5–40 pmol)	[ 17]
IPRPLC-MS/MS	None	--	>31	3 × 10^−10^–9 × 10^−6^	--	[ 53]
IPUPLC-MS/MS	None	--	30	< 5 × 10^−6^	< 1 × 10^−5^	[ 18]
				(10–75 fmol)		
HILIC-MS	None	--	88	3 × 10^−9^–4 × 10^−8^	1 × 10^−8^–1 × 10^−7^	[ 21]
HILIC-MS/MS	None	--	19	1 × 10^−10^–1.2 × 10^−8^	4 × 10^−10^–4.1 × 10^−8^	[ 52]
				(0.6–62 fmol)	(2.1–206 fmol)	
HPLC-MS/MS	Butanol	--	20	1 × 10^−9^–3 × 10^−9^	--	[ 22]
				(15–45 fmol)		
HPLC-MS/MS	FMOC	--	20	5 × 10^−10^–5 × 10^−9^	--	[ 22]
				(7.5–75 fmol)		
HPLC-MS/MS	PrCl	--	20	5.0 × 10^−10^–2.0 × 10^−9^	--	[ 22]
				(7.5–30 fmol)		
HPLC-MS/MS	APDS	--	12	--	3 × 10^−7^–9.3 × 10^−6^	[ 36]
HPLC-MS	APDS	--	13	4 × 10^−8^–2.3 × 10^−6^	--	[ 38]
HPLC-MS/MS	TAHS	--	30	5 × 10^−11^–3.4 × 10^−10^	--	[ 37]
HPLC-MS/MS	iTRAQ®	--	16	5 × 10^−7^–1 × 10^−5^	--	[ 39]
				(1–20 pmol)		
HPLC-MS	AQC	--	50	2 × 10^−7^–6 × 10^−7^	--	[ 50]
UPLC-MS/MS	AQC	--	10	1.02 × 10^−11^–1.06 × 10^−8^	--	This study
				(10.2 amol–10.6 fmol)		

LOD: limit of detection; LOQ: limit of quantitation; AT: Analysis time (time at which all analytes are separated); TCT: total cycle time (*i.e.*, run-to-run time); * time for column reconditioning and re-equilibration is not given, therefore total time of LC gradient is unknown; FMOC: 9-fluorenylmethyl choroformate; PrCl: propyl chloroformate; APDS: 3-aminopyridyl-*N*-hydroxysuccinimidyl carbamate; TAHS: *p-N,N,N*-trimethylammonioanilyl *N’*-hydroxysuccinimidyl carbamate iodide; AQC: 6-aminoquinolyl-*N*-hydroxysuccinimidyl carbamate

### 2.3. Method Application to Screening of Arabidopsis Mutants

Currently, metabolomic studies require high-throughput and sensitive targeted analytical platforms for screening of a large number of genetic variants. Thus, after its evaluation, our AccQ•Tag-UPLC-ESI-MS/MS method was used for the quantitative amino acid determination in *A. thaliana* leaf extracts (9 wild-type samples and 75 mutants; 6 biological replicates each; 504 samples in total) to demonstrate its applicability as a targeted approach for metabolomics analysis (for complete list of *A. thaliana* mutant stocks used in this study refer to Ref. 7). Our method is among the seven analytical platforms employed by the Arabidopsis metabolomics consortium [54] that aims to evaluate the power of using a combination of untargeted metabolomics platforms and targeted profiling methods for key metabolite sets in order to identify the function of GUFs.

**Figure 2:**
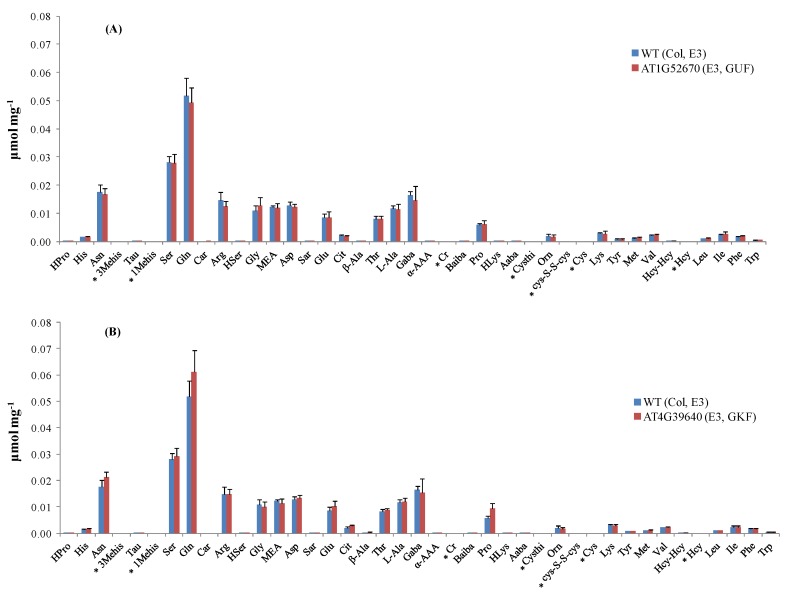
Amino acid profiles in Arabidopsis mutant stocks carrying T-DNA mutant alleles in GKF and GUF. (**A**) Concentration of amino acids (μmol mg^−1^ dry weight) in mutant line SALK_021108 compared to its parental strain (wild-type). (**B**) Concentration of amino acids (μmol mg^−1^ dry weight) in mutant line SALK_004694 compared to its parental strain (wild-type). Standard deviation bars calculated from six biological replicates. For information about Arabidopsis mutant stocks used in experiments E2 and E3 refer to ref. [7]. * Amino acids below LOD. GKF: gene of known function, GUF: gene of unknown function. Hydroxyproline (HPro), histidine (His), asparagine (Asn), taurine (Tau), 1-methyl-histidine (1-Mehis), serine (Ser), glutamine (Gln), carnosine (Car), arginine (Arg), homoserine (HSer), glycine (Gly), ethanolamine (MEA), aspartic acid (Asp), sarcosine (Sar), glutamic acid (Glu), citrulline (Cit), β-alanine (β-Ala), threonine (Thr), L-alanine (L-Ala), γ-amino-n-butyric acid (Gaba), α-aminoadipic acid (α-AAA), creatinine (Cr), β-aminoisobutyric acid (Baiba), proline (Pro), hydroxy-lysine (HLys), α-amino-n-butyric acid (Aaba), cystathionine (Cysthi), ornithine (Orn), cystine (Cys-S-S-cys), cysteine (Cys), lysine (Lys), Tyrosine (Tyr), methionine (Met), valine (Val), homocystine (Hcy-Hcy), homocysteine (Hcy), leucine (Leu), isoleucine (Ile), phenylalanine (Phe), and tryptophan (Trp).

**Figure S3:**
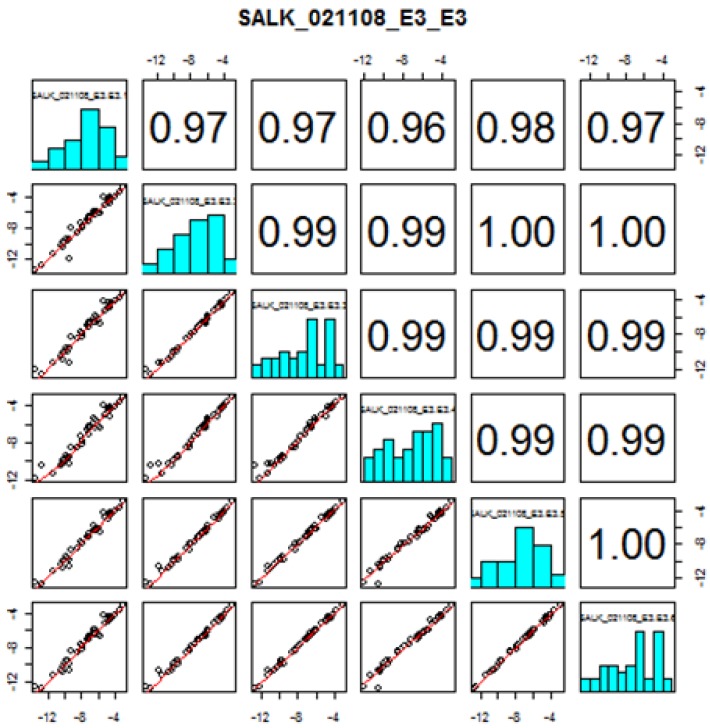
Replicate quality analysis plot for the amino acid profiling by UPLC-LC-MS/MS in the mutant stock SALK_021108 (AT1G52670). Instructions in how to interpret this plot can be found in the consortium web portal (Figure taken from www.PlantMetabolomics.com). The numbers in the upper right corner correspond to the correlation coefficients between replicates (ith row, jth column). The x (ith replicate) and y (jth replicate) coordinates of the scatterplots are the logarithms (base 2) of the ratio of the mean relative abundances (μ) of each amino acid in the wild-type (wt) *versus* mutant (mt) plant (*i.e.*, log_2_ (μ_mt_/u_wt_)).

Thirty-five out of the 38 targeted amino acids were identified and detected above their LODs in the *A. thaliana* leaf extracts. Figure 2 shows the amino acid profiles of two mutant stocks carrying T-DNA mutant alleles in genes of known function (GKFs) and GUFs. Quantitation was based on relative peak areas (as response) of each compound using the calibration curves that were constructed employing the internal standard method. Asparagine (Asn), serine (Ser), glutamine (Gln), arginine (Arg), glycine (Gly), ethanolamine (MEA), aspartic acid (Asp), threonine (Thr), L-alanine (L-Ala), γ-amino-n-butyric acid (Gaba), proline (Pro), lysine (Lys), valine (Val), isoleucine (Ile) were among the most abundant amino acids in the extracts. 3-Methyl-histidine (3-Mehis), 1-methyl-histidine (1-Mehis), creatinine (Cr), cystathionine (Cysthi), cystine (Cys-S-S-cys), cysteine (Cys), and homocysteine (Hcy) were not detected (below LOD) in any of the samples studied (wild-type and mutants). Details on the statistical data processing were already published in two previous papers by the Arabidopsis Metabolomics Consortium [1,7] and will not be covered in this paper. Data quality check performed to determine the variability in amino acid concentration between different biological replicates showed correlation coefficients between 0.61–1.00. Correlation coefficients were < 0.7 in the majority of the cases, indicating the high reliability between the replicates obtained with our amino acid profiling platform. Figure S3 shows the data quality plot for the analysis of amino acids in the mutant SALK_021108 (AT1G52670). Data quality plots for all the mutants analyzed with our AccQ•Tag-UPLC-MS/MS platform can be found in the web portal of the consortium [54].

It is obvious that the amino acid profiling alone is not enough to represent the metabolic effect of gene knockout in the group of T-DNA mutants stocks selected in the initial three metabolomic experiments (E1, E2 and E3) and, therefore, interpretation of the biological significance of the data is outside the scope of this manuscript. However, the combination of our AccQ•Tag-UPLC-ESI-MS/MS platform with other targeted and untargeted method gives a more holistic view of changes in the metabolome. The statistically evaluated data compiled by the consortium of laboratories (including our research group) is publicly available through the web-based project database [54] in order to incentive its use by the metabolomics community for the formulation of hypothesis about the function of GUFs. A discussion of exemplary datasets was already published elsewhere by members of the consortium [7].

## 3. Experimental Section

### 3.1. Chemicals and Reagents

The L-amino acids kit (Sigma-Aldrich, Co., St. Louis, MO, USA) was used for direct infusion experiments and a commercial mix of amino acids and related compounds (Sigma-Aldrich, Co., St. Louis, MO, USA) was employed in the preparation of calibration standards. Asparagine, glutamine and homoserine were purchased separately (Sigma-Aldrich, Co., St. Louis, MO, USA) since they are not included in the commercial mix. Stable- isotope-labeled reference compounds (L-asparagine-^15^-N_2_; L-serine,2,3,3-d_3_; L-glutamine-2,3,3,4,4-d_5_; glycine-d_5_; D-L-alanine-2,3,3,3-d_4_; proline-2,5,5-d_3_; methionine-methyl-d_3_; tryptophan-2',4',5',6',7'-d_5_(indole-d_5_); leucine-d_10_; valine-d_8_; L-histidine (ring 2-^13^C); L-glutamic acid-2,4,4-d_3_; ornithine-3,3,4,4,5,5-d_6_; lysine-3,3,4,4,5,5,6,6-d_8_; phenyl-d_5_-alanine; 4-hydroxyphenyl-2,6-d_2_-alanine-2-d_1_) were used as internal standards and were purchased from Cambridge Isotope Laboratories (Andover, MA, USA) and CDN isotopes (Pointe-Claire, Quebec, Canada). The AccQ•Tag Ultra derivatization kit (AccQ•Tag Ultra borate buffer, AccQ•Tag Ultra reagent powder, and AccQ•Tag Ultra reagent diluent) was obtained from Waters Corporation (Milford, MA, USA). AccQ•Tag Ultra eluents for UPLC-ESI-MS/MS analysis were also from Waters. Ammonium acetate was purchased from Fisher (Fair Lawn, NJ, USA); ammonium hydroxide was supplied by Sigma (St. Louis, MO, USA). LC-MS grade methanol was purchased from J.T. Baker (Phillipsburg, NJ, USA). Ultrapure water (18.2 MΩ-cm) was obtained from a MilliQ Ultrapure water system (Millipore, Bedford, MA, USA). Ultra high purity argon and nitrogen gas for mass spectrometric analysis were purchased from Speciality Gases (Radnor, PA, USA).

### 3.2. Plant Material

Seed stocks of Arabidopsis thaliana mutants were obtained from ABRC and propagated by the central lab of the Arabidopsis Metabolomics Consortium at Iowa State University. This paper focuses on the results obtained by targeted amino acid analysis on leaf extracts of 69 mutant lines selected for three metabolomic experiments (E1, E2, and E3) designed by the consortium. Six biological replicates of each mutant line were provided along with control samples (Columbia (Col-0) ecotype) for each metabolomic experiment.

The list of T-DNA knock-out mutants, the rationale for their selection, plant growth conditions, and protocol for plant harvesting are published elsewhere [1,7] and also available in the project database [54]. Plant material was stored at −80 °C upon arrival.

### 3.3. Amino Acid Extraction from Arabidopsis Samples

Amino acids were extracted from 5 mg (dry weight) of Arabidopsis leaf sample with 125 μL of 50% (v/v) methanol:water solution spiked with isotopically labeled internal standards at 4 μg/mL. Samples were grinded in a mixer mill for 60 sec, incubated on dry ice for 5 min, and sonicated in a water bath for 1 min. Two cycles of buffer extraction, grinding, dry ice incubation, and sonication were completed. At the end of each cycle, the debris was removed by centrifugation at 13 K rpm, 4°C, and 8 min in a Beckman-Coulter refrigerated benchtop centrifuge. The extract was transferred each time to a limited volume vial.

### 3.4. Accq•Tag Ultra Amino Acid Derivatization

The AccQ•Tag Ultra derivatization kit (Waters Corp.) was used in all derivatization procedures, unless otherwise noted. AccQ•Tag Ultra borate buffer was replaced with the ammonium acetate buffer only for direct infusion mass spectrometry experiments. Following the protocol provided by the manufacturer, 10 μL of either a standard amino acid mix solution or an *Arabidopsis* leaf extract was mixed with 70 μL of AccQ•Tag Ultra borate buffer (pH = 8.8). The derivatization was carried out by adding 20 μL of reconstituted AccQ•Tag Ultra reagent (3 mg/mL of AQC in acetonitrile) to the buffered mixture. The sample was immediately vortexed followed by incubation for 15 min at 55 °C.

To maintain consistency between the time of extraction and time of analysis due to the large-scale of the project, derivatized samples were prepared and analyzed by UPLC-ESI-MS/MS in daily batches.

### 3.5. UPLC-ESI-MS/MS Analysis

UPLC-ESI-MS/MS analysis was carried out on a Waters Acquity UPLC system on-line coupled to a Waters Xevo TQ mass spectrometer by means of an electrospray ionization (ESI) probe. Derivatized amino acids were separated on a Waters AccQ•Tag Ultra column (2.1 mm i.d. × 100 mm, 1.7 μm particles). The separation gradient used was: 0–0.54 min (99.9% A), 5.74 min (90.0% A), 7.74 min (78.8% A), 8.04–8.64 min (40.4% A), 8.73–9.50 min (99.9% A). The working eluent A was 10% AccQ•Tag Ultra concentrate solvent A in ultrapure water (Eluent A concentrate composition: acetonitrile (10%), formic acid (6%), ammonium formate in water (84%)), eluent B was 100% AccQ•Tag Ultra solvent B (acetonitrile), and the column flow rate was 0.7 mL/min. The autosampler temperature was set at 25 °C and the column temperature at 55 °C. The sample injection volume was 1 μL.

MS method development started with the direct infusion of individual AQC-derivatized amino acids (1 × 10^−2 ^g/L) into the ESI source of the mass spectrometer at the default infusion rate (20 μL/min). MRM transitions with their respectively optimized cone voltage and collision energy values were determined for each metabolite using the Waters IntelliStart software. The common main product from the collision-induced dissociation of all the AQC adducts was the ion m/z 171, derived from the cleavage at the ureide bond formed upon derivatization. Using the MS parameters fine-tuned by IntelliStart, derivatized standard amino acid solutions (25 μM) were injected into the UPLC-ESI-MS/MS system to determine their retention times. 

The final MRM-MS method employed for the quantitation of amino acids and internal standards was composed of 53 ESI+ timed functions properly segmented over the 10 min chromatographic run. The time segment of each function was selected based on the retention times observed for the metabolites and reference compounds, and ranged from 0.42 to 1.03 min. To increase the overall performance, the MRM-MS method was built to monitor only one transition channel per MRM function. The most sensitive parent-daughter ion transition of each derivatized amino acid (*i.e.*, m/z [M-H]^+^ > 171) was selected for quantitation. 

The following ionization source settings were used: capillary voltage, 1.99 kV (ESI+); desolvation temperature, 600 °C; desolvation gas flow rate, 1000 L/h; source temperature, 150 °C. The analyzer settings were as follows. For quadrupole 1, the low mass resolution was 2.91387 and the high mass resolution was 15.1501; while for quadrupole 2, the values were 2.97214 and 14.7422, respectively. Argon was used as collision gas at a flow rate of 0.15 mL/min.

The UPLC-ESI-MS/MS system control and data acquisition were performed with the Waters Corporation MassLynx^TM^ software. Data analysis was conducted with the TargetLynx^TM^ software (Waters Corporation).

### 3.6. UPLC-ESI-MS/MS Method Evaluation and Applicability

Method evaluation involved the determination of linearity (regression coefficient and dynamic range), sensitivity (detection limits), and reproducibility (relative standard deviations of retention times and peak areas) of the analysis for each amino acid. Working standards with concentration range from 250 μM to 476.8 pM were prepared by serial dilutions of a 500 μM amino acid mix solution spiked with isotopically labeled internal standards at 4 × 10^−3^ g/L. The serial dilutions were performed in a Biomek 2000 Beckman Coulter laboratory automation workstation (Fullerton, CA) using a solution containing the internal standards at 4 × 10^−3^ g/L in a 50% (v/v) methanol:water mixture in order to keep their concentration constant. After derivatization the concentrations of amino acids were decreased 10-fold and the concentration of all internal standards was maintained constant at 4 × 10^−4^ g/L. Calibration curves were obtained by replicate injection of each of the derivatized working standards and were constructed as plots of relative peak area (Area _amino acid_/Area _internal standard_) *versus* amino acid concentration using the TargetLynx software. The assignments of internal standards are given in Table S4.

The applicability of the UPLC-ESI-MS/MS method for sensitive throughput analysis of amino acids was evaluated by determination of their concentrations in derivatized *Arabidopsis thaliana* leaf extracts obtained as described in numeral 3.3 and 3.4.

## 4. Conclusions

An AccQ**•**Tag-UPLC-ESI-MS/MS method that uses stable-isotope-labeled internal standards and scheduled MRM functions was presented for reliable and sensitive quantitation of amino acids. The major advantage offered by this method was the enhanced sensitivity for the analysis, which allowed detection of amino acids at concentration levels down to 1.02 × 10^−11^ M (*i.e.*, 10.2 atomole on column). This latest method represents an improved sensitivity for amino acid analysis of 1 to 5 orders of magnitude compared to existing methods. The AccQ**•**Tag-UPLC-ESI-MS/MS method was successfully applied to the analysis of 504 Arabidopsis leaf extracts and could be easily implemented for the analysis of amino acids under a typical work flow for metabolomics research. The analysis of the plant extracts by the AccQ•Tag-UPLC-ESI-MS/MS method was completed with minimum column care, high repeatability, and reproducible separation which is in sharp contrast to existing HILIC and IPRPLC approaches. Contrary to a common misconception with respect to precolumn derivatization methods, the AQC derivatization worked well for all the amino acids tested and the AccQ**•**Tag-UPLC-ESI-MS/MS method gave reliable data for metabolomic studies.

## Appendix

**Table S1:** Reproducibility of peak areas for AQC-derivatives of isotopically labeled amino acid standards obtained in 50 mM ammonium acetate buffer (pH 9.3). Experimental conditions were the same as described in Section 3.5.

Isotopically labeled amino acid	Area	Standard deviation	RSD (%)
L-Asparagine-^15^-N_2_	37623	307	8.15
L-Serine,2,3,3-d_3_	4902	407	8.29
L-Glutamine-d_5_	2453	172	7.02
Glycine-d_5_	6450	418	6.47
Threonine-d_5_	6202	496	7.99
D-L-Alanine-2,3,3,3-d_4_	2405	211	8.78
Proline-2,5,5-d_3_	2182	191	8.74
4-Hydroxyphenyl-2,6-d_2_-alanine-2-d_1_-01	7152	588	8.23
Methionine-methyl-d_3_	7254	479	6.60
Tryptophan-2',4',5',6',7'-d_5_(indole-d_5_)-01	7224	318	4.40

**Table S2:** Long term repeatability of retention times for AQC-derivatized amino acids in standard solutions analyzed by UPLC-ESI-MS/M (n = 30).

Amino acid	Retention Time			
	Intra-day results, day 1		Intra-day results, day 47	
	Average (min)	RSD (%)	Average	RSD (%)
Hydroxyproline	1.49	1.04	1.52	0.75
Histidine	1.60	1.22	1.65	1.04
Asparagine	1.84	0.71	1.89	0.58
3-Methyl-histidine	1.97	0.83	2.03	0.65
Taurine	2.13	0.57	2.17	0.48
1-Methyl-histidine	2.20	0.71	2.25	0.69
Serine	2.51	0.46	2.55	0.34
Glutamine	2.67	0.46	2.71	0.28
Carnosine	2.74	0.45	2.77	0.36
Arginine	2.77	0.58	2.81	0.29
Glycine	2.88	0.35	2.92	0.25
Homoserine	3.02	0.34	3.04	1.30
Ethanolamine	3.04	0.31	3.06	0.39
Aspartic acid	3.24	0.32	3.27	0.19
Sarcosine	3.68	0.19	3.69	0.25
Glutamic Acid	3.84	0.25	3.86	1.54
Citrulline	3.87	0.22	3.89	0.13
β-Alanine	4.08	0.19	4.09	0.16
Threonine	4.30	0.15	4.31	0.08
L-Alanine	4.74	0.15	4.75	0.09
γ-Amino-n-butyric acid	4.92	0.10	4.92	0.03
α-Amino adipic acid	5.13	0.13	5.13	0.07
β-Aminoisobutyric acid	5.38	0.16	5.38	0.08
Proline	5.39	0.10	5.39	0.00
α-Amino-n-butyric acid	5.99	0.10	5.99	0.07
Tyrosine	6.61	0.10	6.61	0.03
Methionine	6.77	0.09	6.76	0.00
Valine	6.91	0.04	6.90	0.05
Leucine	7.67	0.04	7.67	0.03
Isoleucine	7.75	0.02	7.75	0.02
Phenylalanine	7.86	0.03	7.86	0.00
Tryptophan	7.96	0.05	7.97	0.06

**Table S3:** Assignment of internal standards for UPLC-ESI-MS/MS determination of AQC-amino acid derivatives.

Compound number	Amino acid	Internal standard
1	Hydroxyproline	31
2	Histidine	3
3	L-Histidine (ring 2-^13^C)	
4	Asparagine	5
5	L-Asparagine-^15^-N_2_	
6	3-Methyl-histidine	3
7	Taurine	11
8	1-Methyl-histidine	3
9	L-Serine-2,3,3-d_3_	
10	Serine	9
11	L-Glutamine-2,3,3,4,4-d_5_	
12	Glutamine	11
13	Carnosine	3
14	Arginine	36
15	Glycine-d_5_	
16	Glycine	15
17	Homoserine	9
18	Ethanolamine	15
19	Aspartic Acid	21
20	Sarcosine	15
21	L-Glutamic acid-2,4,4-d_3_	
22	Glutamic Acid	21
23	Citrulline	11
24	β-alanine	15
25	Threonine	9
26	D-L-alanine-2,3,3,3-d_4_	
27	L-Alanine	26
28	γ-Amino-n-butyric acid	15
29	α-Amino adipic acid	21
30	β-Aminoisobutryic acid	15
31	Proline-2,5,5-d_3_	
32	Proline	31
33	δ-hydroxylysine	39
34	α-Amino-n-butyric acid	26
35	Cystathionine	37
36	Ornithine-3,3,4,4,5,5-d_6_	
37	Ornithine	36
38	Cystine	36
39	Lysine-3,3,4,4,5,5,6,6-d_8_	
40	Lysine	39
41	Tyrosine	50
42	Methionine-methyl-d_3_	
43	Methionine	42
44	Valine-d_8_	
45	Valine	44
46	Homocystine	36
47	Leucine	49
48	Isoleucine	49
49	Leucine-d_10_	
50	Phenyl-d_5_-alanine	
51	Phenylalanine	50
52	Tryptophan-2',4',5',6',7'-d_5_(indole-d_5_)	
53	Tryptophan	52

**Table S4:** Overall process efficiency (PE%) of the AQC derivatized internal standards.

	Intraday assay^a^		Interday assay^b^	
Internal standard	PE%	CV%	PE%	CV%
L-Histidine (ring 2-^13^C)	77.4	5.2	73.9	6.9
L-Asparagine-^15^-N_2_	70.9	5.0	68.8	6.5
L-Serine-2,3,3-d_3_	71.1	4.5	69.4	5.6
L-Glutamine-2,3,3,4,4-d_5_	65.2	5.1	65.0	5.6
Glycine-d_5_	79.6	5.0	76.7	5.9
L-Glutamic acid-2,4,4-d_3_	83.1	5.2	79.7	6.7
D-L-alanine-2,3,3,3-d_4_	86.8	5.2	81.8	7.3
Proline-2,5,5-d_3_	97.0	4.0	99.4	8.3
Ornithine-3,3,4,4,5,5-d_6_	84.5	5.5	77.3	9.4
Lysine-3,3,4,4,5,5,6,6-d_8_	85.6	26.7	98.1	18.9
Methionine-methyl-d_3_	97.1	3.5	85.3	11.5
Valine-d_8_	90.6	4.9	92.5	9.3
Leucine-d_10_	94.3	5.1	94.6	10.4
Phenyl-d_5_-alanine	94.3	5.3	94.9	10.1
Tryptophan-2',4',5',6',7'-d_5_(indole-d_5_)	99.4	3.3	89.5	10.2

^a^ Intraday essays were calculated in 20 *Arabidopsis* leaf extracts analyzed the same day as the calibration standards; ^b^ Interday essays were calculated in 60 *Arabidopsis* leaf extracts analyzed one day after the calibration standards.
